# Allied health professionals’ perceptions of research in the United Kingdom national health service: a survey of research capacity and culture

**DOI:** 10.1186/s12913-022-08465-6

**Published:** 2022-08-27

**Authors:** Christine Comer, Richard Collings, Alison McCracken, Carol Payne, Ann Moore

**Affiliations:** 1 Leeds Community Healthcare NHS Trust, Stockdale House, Headingley Office Park, 8 Victoria Road, Leeds, UK; 2 Faculty of Medicine, University of Leeds, Leeds, UK; 3 Torbay and South Devon NHS Foundation Trust, Castle Circus Health Centre, Torquay, UK; 4 University Hospitals of Morecambe Bay NHS Foundation Trust, Kendal, UK; 5 Norfolk and Norwich University Hospitals NHS Foundation Trust, Norwich, Norfolk, UK; 6 Professor Emerita, University of Brighton, Brighton, UK

**Keywords:** Allied health professions, Survey, Research capacity, Research culture

## Abstract

**Background:**

With growing recognition of the importance of research in allied healthcare, the new Health Education England (HEE) research strategy articulates a need to transform Allied Health Professional (AHP) identities, culture and roles. An understanding of current AHP research capacity and culture is first required.

**Methods:**

A cross-sectional survey targeted AHPs working in NHS health and social care settings across the United Kingdom. The validated Research Capacity and Culture tool was modified and distributed through research and professional networks. Aggregate median scores for perceived research success were categorised as adequate, more than, or less than adequate.

**Results:**

Of 3344 participants, 3145 identified as HEE-defined AHPs. Individual- and organisation-level research success was perceived as adequate (median scores 4 (IQR 2 to 6); 4 (IQR 2 to 7) respectively). Team-level research success was rated less than adequate (median score 2 (IQR 1–5)).

**Conclusions:**

In the UK, AHPs working in NHS health and social care perceive individual and organisational level research skill/success to be adequate. In contrast, inadequacies in research skill/support at team level were exposed, which may hinder successful integration of allied health research into everyday health and social care practice. Recommendations are made with reference to the HEE AHP research strategy.

**Supplementary Information:**

The online version contains supplementary material available at 10.1186/s12913-022-08465-6.

## Introduction

Research is fundamental to health care. A culture of research promotes the expectation that everyday health and social care is based on best available knowledge and research evidence [1]. Healthcare organisations with a strong research culture identify and develop research questions relevant to their specific healthcare setting and the population they serve [2], and they understand the importance of generating new knowledge to contribute to the research evidence base [3]. As a result, research active organisations provide superior health service performance, higher quality of care, improved patient safety, and a better patient experience [4–6]. Furthermore, they are able to provide greater opportunities for staff development [7]. In such organisations, research achievements are recognised, there is investment of resources to support research activity, and clinicians are encouraged to and are given the opportunity to develop skills and participate in research-related activities [6].

All healthcare professions (not just the medical and nursing professions who have traditionally been seen in research roles) are now expected to take an active role in informing, supporting, delivering, implementing and leading clinical research. This expectation is increasingly driven by national healthcare policies and frameworks: The UK Policy Framework for Health and Social Care Research stipulates that research is ‘a core function of health and social care’ [8], and the National Health Service (NHS) Long Term Plan identifies research as a key driver for all professions to improve future health outcomes [9]. More explicitly, the Department of Health and Social Care (DHSC) sets out a vision for clinical research to be embedded in the NHS, where a research-positive culture ensures that all health and care staff feel empowered to support and participate in clinical research as part of their job [10]. Despite this broad vision, no clear benchmark has been established to define a research-positive culture in the AHP workforce. Whilst AHP research activity is noticeably less than in the medical and nursing professions, little is known about how AHPs working across different health and social care settings perceive their current research capacity and culture.

AHPs represent the third largest professional group working in the NHS [11]. Currently, 220,000 Allied Health Professionals (AHPs) are registered to practice across the United Kingdom (UK), and over 90,000 of these work in the NHS in England alone [12]. The AHP Research Strategy for 2018–2020 produced by the National Institute for Health and Care Research (NIHR) Clinical Research Network, documented the vital role AHPs can play in the delivery of high-quality patient-centred clinical research [13]. To improve research readiness in the AHP workforce, the Council of Deans of Health advocates for stronger AHP clinical academic career pathways [14] and HEE stipulates a requirement for research to be one of the four pillars underpinning Advanced Clinical Practice across all health and care professions [15].

High quality AHP-led research is needed more than ever to underpin new models of healthcare delivery and to inform AHP roles that are rapidly evolving to meet the changing needs of the population. Without appropriate research, advancing AHP roles may lack the evidence required to attract resources and to optimise effective care pathways for patients. However, with few senior clinical academic AHP leaders and decision-makers [16] and little focus on building AHP research capacity in NHS health and social care, there are considerable challenges for allied health research. Research growth is hindered by low numbers of AHPs working in clinical academic roles to provide much needed leadership [17]. Fellowship awards aimed at building research capacity and leadership by developing clinical academics are available through the NIHR. However, a review of these highly competitive schemes found disparities in gaining awards between AHP professional groups, as well as limited uptake of more senior fellowships at postdoctoral level [18]. Research capacity-building frameworks suggest, therefore, that system-wide, sustained change is needed to address challenges at organisation-, team- and individual-level [16, 19–21].

This need for system-wide change is reflected in the recently launched AHP-specific research strategy developed by HEE [22]. The strategy sets out its multidimensional aims to i) transform AHP professional identities, culture and roles; ii) deliver excellence in research and practice; and iii) ensure inclusion of allied health research and innovation in National strategic research agendas. To inform the implementation and to evaluate the future impact of this strategy, an understanding of current AHP research capacity and culture is required.

## Methods and materials

The aim of this study is to generate a UK-wide picture of the perceived level of research capacity and culture within AHP professions working in NHS health care and social care.

The validated Research Capacity and Culture (RCC) questionnaire [21] was selected as the best available tool to comprehensively measure perceptions of research capacity and culture through self-reported ratings of research success/skill across a range of individual, team- and organisation-level research constructs [23]. Previously published studies using the RCC tool in the UK have targeted relatively small samples of AHPs from a single healthcare organisation [24–26]. To our knowledge, this study represents the first to provide a UK-wide perspective across all AHP professions working in health and social care in the UK, exploring their perceptions of the levels of support for research at an organisational and team level as well as their own level of research skill and confidence. The project proposal was classified through the Health Research Authority (HRA) automated system as not requiring ethical approval (IRAS 277,676). Health services research permission was provided by HRA and Care Research Wales (HCRW) (REC 21/HRA/0053), and the study was adopted onto the NIHR portfolio (CPMS ID 47,506). The study was performed in accordance with ethical standards laid down in the 1964 Declaration of Helsinki and its later amendments. Informed consent was gained from all participants before completion of the survey.

Specific objectives for this study were to:

- gauge perceptions of AHP research support and capacity at organisation and team level
- gauge participants’ perceptions of their own research skills/knowledge and confidence
- identify key perceived barriers and motivators for research engagement

### Study design

A national cross-sectional survey was conducted, targeting AHPs working in all NHS health and social care settings.

The online survey was distributed electronically via NIHR Clinical Research Network (CRN) health services research networks in England, health boards in Scotland, Northern Ireland and Wales, and through AHP professional and research bodies. The survey remained open from its launch on 01–06-21 to closure on 30–09-21 and was publicised intermittently during this period via NIHR CRN channels, AHP professional bodies including Health and Care Professions Council (HCPC), and research organisations including Council for Allied Health Professions Research (CAHPR) via email, written bulletins and social media channels.

### The survey tool

The RCC tool [27] is a questionnaire designed to measure indicators of research capacity and culture at individual-, team- and organisation-level. It has been shown to be reliable and valid in AHP populations [21]. Research success/ skill level is ranked for each of 48 items on a scale of 1–10. Further questions addressing factors such as barriers and facilitators to research, current research activity and experience use multiple choice options. Free-text response options are offered for participants to provide supplemental details or comments if desired.

For this study, the survey questionnaire (available in supplementary materials 1) was modified by the addition of questions that focused on self-reported research engagement level; discussion of research during appraisals; time allocated for research for those who indicated that research was part of their role description; and awareness of national-level research organisations. The survey also integrated a 6-point scale to determine respondents’ self-assessment of their current attainment level in clinical research skills [28]. This newly developed ‘Skills, Capability, and Organisational Research Readiness’ (SCORR) scale has been recommended for use as an appraisal tool for non-medical registered healthcare professionals working within healthcare.

Demographic data collected from participants in the survey included geographical area of work, professional background, educational history and research experience. No personally identifiable information was collected, and to avoid participant identification during data analysis, no detailed employer details were requested.

### Participants and recruitment

AHPs and other healthcare professionals regulated by the Health and Care Professions Council (HCPC), who were working in NHS health care and local authority social care settings in the UK at the time of completing the survey were eligible to participate. The wider allied health workforce governed by HCPC (Clinical Scientists, Biomedical Scientists, Practitioner Psychologists, and Hearing Aid Dispensers, marked below with an asterisk), but not included on the HEE list of AHPs, were also eligible to participate. Eligibility criteria were defined on the opening page of the survey for potential participants as follows:

- a qualified/registered Allied Health Professional (AHP) from the following list: Art therapist, Music therapist, Drama therapist, Biomedical scientist*, Chiropodist/ podiatrist, Clinical Scientist*, Dietitian, Hearing aid dispenser*, Operating department practitioner, Orthoptist, Occupational therapist, Osteopath, Paramedic, Physiotherapist, Prosthetist/ Orthotist, Practitioner psychologist*, Radiographer, Speech and language therapist
- currently working in the NHS, local authority, or an organisation providing NHS-funded healthcare in the UK (England, Scotland, Wales or Northern Ireland)

Before accessing the survey, potential participants were asked to confirm their eligibility, and that they had read and understood the study information provided on the opening page. They were required to provide informed consent prior to accessing and completing the survey questionnaire. In addition, they were asked to state whether or not they wished their anonymised data to be included in shared data for future research/ strategy development. After piloting within the study team, the survey was estimated to take around 20 min to complete.

### Analysis

Quantitative data were analysed using IBM SPSS Statistics software version 25. Likert-scale items within the RCC tool and additional questions were summarised in accordance with convention for ordinal data using the median and Inter Quartile Range (IQR) for each item within the individual-, team-, and organisation-level domains. Aggregate median scores that combined the scores of all items within each domain were categorised in line with previously published literature [29], in which scores lower than 4 are interpreted as less than adequate; scores between 4.0 to 6.99 are interpreted as adequate, and scores greater than 6.99 are interpreted as more than adequate. Frequencies and percentages of responses were used to evaluate ‘Unsure’ and ‘Not applicable’ response categories for these items, and for all other categorical questionnaire items.

Free text items were analysed by inductive content analysis [30]. This included open coding of the narratives and grouping into subcategories. The qualitative data analysis software NVivo (v12.0) facilitated the organization and structuring of the process of coding and grouping and the development of relationships among concepts.

## Results

A total of 3344 participants indicated their eligibility and completed the survey, of whom 3276 agreed for their responses to be included as part of an anonymised open-access data set.

### Study participants

Participants included 3145 from the 14 HEE listed AHPs. A further 127 participants were from healthcare professions who were invited to participate as part of the wider allied health workforce governed by HCPC (Clinical Scientists, Biomedical Scientists, Practitioner Psychologists, and Hearing Aid dispensers) (Table 1). Additionally, 69 respondents completed the survey but indicated within the survey responses that they did not belong to any of these professions, so were excluded from the analyses. For the purposes of our analyses in this manuscript, we included the 3145 participants from HEE listed AHP professions.

**Table 1:** Brief demographic summary of participants

**Profession**	**Number** (n)	**Percentage** (%)
Occupational Therapist	747	22.4
Physiotherapist	1134	33.9
Radiographer (diagnostic and therapeutic)	240	7.2
Podiatrist / Chiropodist	160	4.8
Dietitian	268	8.0
Speech and language therapist	328	9.8
Music therapist	17	0.5
Art therapist	25	0.7
Drama therapist	7	0.2
Prosthetist / Orthotist	33	1.0
Paramedic / Emergency Care Practitioner	70	2.1
Operating Department Practitioner	62	1.9
Orthoptist	49	1.5
Osteopath	5	0.1
Other (including biomedical scientists, healthcare scientists, hearing aid dispensers, and other non HCPC regulated professions, not included in analyses presented in this paper	196	5.9
**Highest level of qualification**		
No formal professional / academic qualification	1	< 0.1
Certificate / Diploma	173	5.5
Degree	1418	45.1
MSc / post-graduate	1363	43.4
PhD	166	5.3
Other	21	0.7
**Type of Healthcare Organisation/ Trust**		
NHS Acute Trust	1659	52.8
NHS Ambulance Trust	43	1.4
NHS Community / Care Trust	900	28.6
NHS Mental Health Trust	306	9.7
GP practice	16	0.5
Primary Care Network	42	1.3
Clinical Commissioning Group	8	0.3
Local authority providing NHS-funded health or social care	30	1.0
Independent provider of NHS-funded healthcare	36	1.1
Other	105	3.3
**Country**		
Total from England	2922	92.9
Total from Scotland	121	3.8
Total from Wales	35	1.1
Total from Northern Ireland	67	2.1
Total from Channel Islands/ Isle of Man	0	0

More detailed demographic detail, including regional response rates, pay-banding, years of experience, and diversity details are available in the main report, supplementary material 2

The majority of participants were physiotherapists, occupational therapists, or speech and language therapists, almost half with post-graduate qualifications (Table 1). Most were from England, with over half working in Acute Hospital Trust settings. Further details are available in supplementary materials 2.

### Research capacity and culture (RCC) tool scores

The aggregate median score for research skill/success at individual-level was 4 (IQR 2 to 6) on a 0–10 scale, representing an ‘adequate’ score (Table 2). Highest levels of skill were reported for finding and critically reviewing literature, and lowest levels in securing research funding. The aggregate median score for research skill/ success at team-level fell below the range classed as adequate (median 2, IQR 1–5) with only two items reaching an adequate score (has team leaders that support research; undertakes planning guided by evidence). For organisation-level research skill/ success, aggregate scores reached an adequate level overall (median 4, IQR 2–7), with highest scores for promotion of evidence based clinical practice, and lowest scores for ensuring staff career pathways are available in research, and having software programs for analysing research data.

**Table 2:** RCC scores (scale 0–10) for individual-, team-, and organisation-level research success/ skills

	Median (IQR)	Unsure (%)
**Individual level**		
Finding relevant literature	7 (5–8)	0.2
Critically reviewing the literature	7 (5–8)	0.3
Using a computer referencing system (e.g. Endnote)	5 (2–7)	1.8
Writing a research protocol	3 (2–6)	1.0
Securing research funding	1 (1–3)	2.4
Submitting an ethics application	2 (1–5)	1.7
Designing questionnaires	4 (2–7)	1.0
Collecting data e.g. surveys, interviews	5 (3–7)	0.9
Using computer data management systems	3 (1–6)	1.7
Analysing qualitative research data	4 (2–6)	0.9
Analysing quantitative research data	4 (2–6)	0.9
Writing a research report	4 (2–7)	1.1
Writing for publication in peer-reviewed journals	2 (1–5)	1.7
Providing advice to less experienced researchers	2 (1–5)	1.3
**Individual level research skills/success overall score**	**4 (2–6)**	**1.2**
**Team level** (2–5% reported they do not work in a team)		
has adequate resources to support staff research training	3 (1–5)	9.7
has funds/equipment/admin to support research activities	2 (1–4)	11.0
participates in team level planning for research development	2 (1–4)	8.5
ensures staff involvement in developing that plan	2 (1–5)	9.2
has team leaders that support research	4 (2–7)	7.2
provides opportunities to get involved in research	3 (1–5)	6.6
undertakes planning that is guided by evidence	4 (2–7)	8.8
has consumer involvement in research activities/planning	2 (1–5)	12.8
has applied for external funding for research	1 (1–4)	14.5
conducts research activities relevant to practice	3 (1–6)	9.4
supports applications for research scholarships/ degrees	2 (1–6)	13.7
has mechanisms to monitor research quality	2 (1–5)	15.6
has identified experts accessible for research advice	2 (1–6)	13.2
disseminates research results at research forums/seminars	2 (1–6)	11.0
supports a multi-disciplinary approach to research	3 (1–6)	10.8
has incentives & support for mentoring activities	2 (1–4)	13.8
has external partners (e.g. universities) engaged in research	3 (1–6)	12.5
supports peer-reviewed publication of research	2 (1–6)	13.6
has software available to support research activities	1 (1–4)	18.1
**Team level research skills/ success overall score**	**2 (1–5)**	**11.6**
**Organisation level**		
has adequate resources to support staff research training	3 (2–6)	19.4
has funds/equipment/admin to support research activities	3 (1–5)	22.5
has a plan or policy for research development	4 (2–7)	24.2
has senior managers that support research	4 (2–7)	16.3
ensures staff career pathways are available in research	2 (1–5)	21.2
ensures organisation planning is guided by evidence	4 (2–7)	19.9
has consumers involved in research	3 (1–6)	27.8
accesses external funding for research	4 (1–7)	27.0
promotes clinical practice based on evidence	6 (3–9)	11.3
encourages research activities relevant to practice	4 (2–7)	15.2
has software programs for analysing research data	2 (1–5)	37.6
has mechanisms to monitor research quality	3 (1–6)	33.8
has identified experts accessible for research advice	4 (1–7)	26.5
supports a multi-disciplinary approach to research	4 (1–7)	22.5
has regular forums/bulletins to present research findings	3 (1–7)	20.2
engages external partners (e.g. universities) in research	4 (1–8)	24.4
supports applications for research scholarships/ degrees	4 (1–7)	26.7
supports the peer-reviewed publication of research	4 (1–7)	28.0
**Organisation level research skills/ success overall score**	**4 (2–7)**	**23.6**

Median (IQR) scores for all 3145 AHP participants. Score range 1 = no success/skill and 10 = highest possible success/skill. *IQR* Interquartile range

### Individual barriers and motivators to research

Barriers and motivators to research on an individual level were explored by both quantitative multiple-choice question as well as free-text boxes. Quantitative data revealed that the key barriers to research engagement were ‘other work roles take priority’ (cited by 83% of respondents) and ‘lack of time for research’ (80%). Whereas primary motivators were ‘to develop skills’ (80%) and ‘increased job satisfaction’ (63%).

Content analysis of the free text responses revealed two categories: enablers and challenges. The enablers category included four subcategories: perceived benefits, funding opportunities, positive support and internal motivation (Table 3). The perceived benefits subcategory emphasised the importance of undertaking research activity to improve patient care and developing an evidence base. Other comments related to the perceived benefits for workforce development and retention whilst improving skills at an individual level. The funding opportunities sub-category highlighted the importance and availability of funding streams as enablers to research activity, although opportunities varied between local (Trust) level and external (National). The sub-category of positive support and culture of the Trust was viewed as being extremely important to enabling research activity, especially when opportunities and encouragement was discussed at appraisals. On an individual basis, a variety of motivational factors for enabling research activity was cited under the sub-category, such as using own initiative to find research and training opportunities, discovering role models to support the journey and linking in with a Higher Educational Institution. The challenges category included four sub-categories: opportunities, system, emotions, and priority (Table 3). The sub-category of opportunities cited the lack of time and limited chances to access research training or pursue academic career pathways as main challenges of undertaking research activity. Interestingly, inadequate research skills and feeling ‘rusty’ from lack of regular involvement in research was also reported by some participants. The sub-category of negative emotions highlighted the feeling of despondency and uncertainty on how to undertake research activity. The sub-category of system factors, such as a lack of research infrastructure and understanding of clinical academic roles and responsibilities alongside the absence of an established career pathway were frequently cited by participants. The priority sub-category highlighted that research activity was perceived as a lower priority in the wider provision of health care, especially in terms of amounts of time, support, finance and expertise that is allocated when compared with other NHS activity.

**Table 3:** Enablers of research and challenges to research

Category	Sub Category	Narrative
**Enablers**	Perceived benefits	*To increase understanding of and exemplify the benefits of my profession for clients* *I think having colleagues who are involved and passionate about research creates an environment which encourages others, particularly newer members of staff to think research could be part of their role. Having management and senior members of the team actively supporting and doing research motivates too*
	Funding opportunities	*External research opportunities can be found and pursued by individuals who are interested in research* *I have had some experience of being funded for research and access to support through the R&D and the host university*
	Support	*We have an AHP research lead, who would support us if we wanted to do research, but any research would be done on top of our existing contract* *but do feel supported by my immediate manager to engage in training and development in research*
	Internal Motivation	*Personal drive to make change, to stretch my mind and answer the constant barrage of questions* *Individual motivation to get involved in audit and research is very high*
**Challenges**	Opportunities	*Our Trust is active in wanting us to pursue clinical academic activity but there is no pathway and no career progression. I have been on the same banding with no chance on promotion* *Apart from mandatory training and diversity work no mention of opportunities for research in academia [and] is rarely advertised on trust intranet*
	System	*Seriously lacks the infrastructure to support research activity* *Infrastructures within the organisation do not exist to support clinical academic pathways and many challenges / barriers exists for those who are seeking active involvement in research*
	Emotions	*Seems overwhelming, time consuming and difficult without support* *My future remains uncertain despite having worked as a clinical academic across the Trust and university for over 7 years*
	Priority	*Workload pressures and short-staffing have limited the time I can spend on research and related activities* *Research is one of the forgotten areas of clinical practice in NHS* *divisional management used the money to reduce overspend rather than provide backfill for hours, and told staff shouldn't be doing research if [there is] no cover for clinical time*

### Research activity, engagement, training/development, and appraisal

Engagement in current research reported most frequently by participants included the use of research evidence to inform clinical practice (85%) and involvement in clinical audit/ research to evaluate/ improve clinical services (64%). The least frequently reported research engagement included taking on roles of Chief Investigator/ research leader (7%) or Site Principal Investigator (8%). Seven percent of participants reported no current engagement in research. The most frequent current or recent research-related activities undertaken by participants were collecting data (25%) and writing/ coauthoring research reports/ publications (16%). The least frequent were applying for (8%) and securing (7%) research funding, and submitting ethics applications (7%). The majority (68%) of participants reported that they had not been involved in any specified research activities over the last 12 months.

Thirty-four percent of participants, reported that research-related activities were part of their role description. Of these, 10% reported that more than 75% of their time was formally allocated for research-related activity, 11% were allocated between 25 and 50% of their time, whilst 79% had less than 25% of their time allocated for research-related activity. In addition, 14% participants reported that they were currently enrolled in further higher degree study or other professional development related to research, of which 72% were undertaking postgraduate diploma or masters level study, and 24% were undertaking PhDs.

**Fig. 1:**
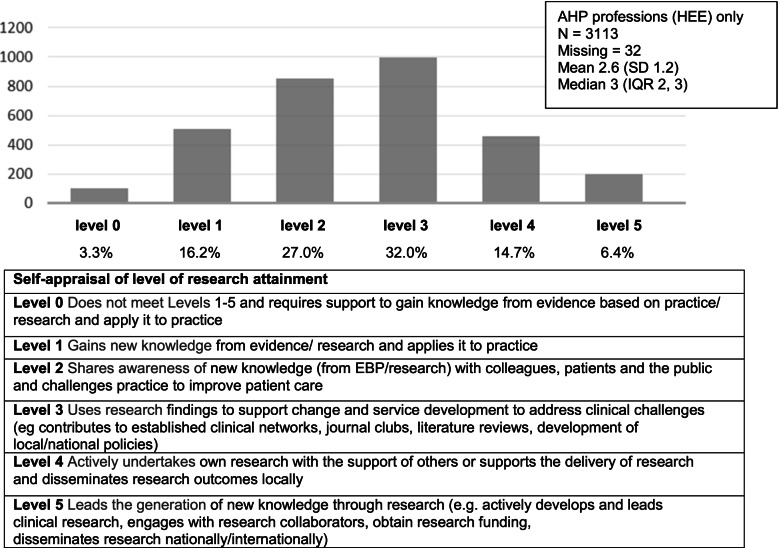
The Clinicians’ Skills, Capability, and Organisational Research Readiness (SCORR) Tool. Levels of research skill defined in the SCORR self-appraisal tool [28]

Eighteen percent of participants reported that research engagement or activity was routinely discussed at their annual appraisal, 50% said that it was only discussed if they brought it up or were currently involved in research, whilst 32% reported that research was not discussed at personal development appraisals on a routine basis. When asked to evaluate themselves on a tool designed to be used during personal development appraisals to rate research engagement, most AHP participants rated themselves as level 2 or level 3 (see Fig. 1), where level 2 indicates that they share awareness of new knowledge from research with colleagues, patients and the public and challenge practice to improve patient care, and level 3 indicates that they use research findings to support change and service development and to address clinical challenges. Twenty-one percent rated themselves as level 4 or 5, indicating actively undertaking, delivering or leading research.

### Awareness of national research support infrastructure

The Council for Allied Health Professions Research (CAHPR) is a UK wide organisation supported by each of the AHP Health Professional bodies. It was launched in 2014 with a mission to ‘develop AHP research, strengthen evidence of the professions’ value and impact for enhancing service user and community care, and enable the professions to speak with one voice on research issues, thereby raising their profile and increasing their influence’. Awareness of CAHPR was low among AHP participants, with 64% reporting that they had little knowledge/ awareness or had never heard of them, with only 32% reporting some/ fairly good knowledge/ awareness.

The National Institute for Health and Care Research (NIHR) was established in 2006 to support health and social care research in and for the NHS. It is funded by the Department of Health and Social Care with a remit to ‘create a health research system in which the NHS supports outstanding individuals, working in world-class facilities, conducting leading-edge research focused on the needs of patients and the public’. Whilst a greater proportion of participants from England reported having ‘some’ or ‘fairly good’ knowledge/ awareness of NIHR (54%), most (74%) had little or no knowledge/ awareness of the NIHR/HEE integrated clinical academic training schemes for AHPs. Levels of awareness of research support infrastructure in England and the devolved nations are presented in Table 4.

**Table 4:** Awareness of research support infrastructure in the UK

	No knowledge/ never heard of them	Heard of them but little knowledge / awareness	Some knowledge/ awareness	Fairly good knowledge/ awareness	In-depth knowledge/ awareness	Not applicable
Council for Allied Health Professions Research (CAHPR) (3145 AHPs across all countries)	983 (31)	1017 (33)	669 (21)	347 (11)	116 (4)	4 (0)
NIHR England (2922 AHPs in England)	281 (10)	781 (27)	931 (32)	656 (22)	260 (9)	7 (0)
Integrated Clinical Academic (ICA) training programmes NIHR/HEE (2922 AHPs in England)	1557 (54)	577 (20)	324 (11)	217 (7)	192 (7)	43 (1)
The Health and Social Care Public Health Agency (HSC PHA) in Northern Ireland (67 AHPs in N.Ireland))	7 (11)	16 (25)	19 (29)	17 (26)	4 (6)	2 (3)
Health and Social Care Northern Ireland (HSCNI) Fellowship Awards (67 AHPs in N.Ireland))	27 (40)	17 (25)	11 (17)	9 (13)	3 (5)	0 (0)
Northern Ireland Clinical Research Network (NICRN) (67 AHPs in N Ireland)	24 (36)	17 (26)	10 (15)	11 (17)	1 (1)	3 (5)
The Chief Scientist Office (CSO) Scotland (121 AHPs in Scotland)	50 (42)	29 (24)	28 (24)	6 (5)	5 (4)	1 (1)
NHS Research Scotland (NRS) career researcher fellowships (121 AHPs in Scotland)	36 (30)	41 (34)	28 (23)	6 (5)	9 (7)	1 (1)
Health and Care Research Wales (35 AHPs in Wales)	10 (29)	10 (29)	8 (23)	2 (5)	5 (14)	0 (0)
Research Capacity Building Collaboration (RCBC) Wales (35 AHPs in Wales)	21 (60)	3 (9)	1 (2)	3 (9)	7 (20)	0(0)

All values represent number of responses (percentage) from participants for whom the question was relevant

## Discussion

This was the first national survey of AHP’s to explore perceptions of research in NHS health and social care. The study fulfilled the aim of generating a UK-wide picture of the perceived research capacity and culture across AHP professions and a range of health and social care settings. The results will provide a benchmark for individual professions and healthcare organisations. A summary of the study and key findings are provided in infographic form in supplementary materials 3.

In contrast to our study in which participants rated research success at team level lower than individual-level or organisation-level, two UK-based studies previously reported team-level research success as high as, or higher than, individual-level ratings. The first of these studies surveyed a small sample of AHPs from a tertiary care hospital and the second surveyed a sample that included both AHPs and nurses from a research focused hospital setting [24, 25]. Notably, the second study also surveyed a sample working in a non-research focused hospital for comparison, and here team-level research success was rated lowest. These contrasting results suggest that differences in research culture may be most apparent at team level. Moreover, the less than adequate team-level scores of research capacity in our results echo anecdotal evidence that blockages to AHP research engagement and activity are particularly evident at middle-management/team level in organisations [24]. Low levels of research confidence among healthcare clinical team managers might account for this, combined with a common perception of conflicting push–pull demands on time and resources between patient care and research. Authors who found a similar disparity between team-level success and success at individual and organisation level in an Australian study [31] concluded that research support at team level does not offer the connection needed between the organisation and the individual. Unfortunately, whilst individual AHPs may feel they have adequate research capability, and whilst research strategies might be produced and endorsed at board level, it falls to middle management to implement such strategies in daily practice and to support research-active individuals within their clinical teams. Team-level ‘middle’ managers therefore have a vital role in implementing evidence-based practice [32] and supporting their clinical teams in performing research activities’ [33]. Our results suggest that survey participants believe that their team leaders do support research. However, only if they are equipped with the appropriate knowledge and skills, resources, authority, and sufficient support from senior management [34] can team-level middle managers effectively operationalize this support to facilitate AHP research engagement [35]. Of note, a recent Australian study surveying multiple healthcare disciplines suggests that inadequate research skill/ support at team-level may be a problem specific to allied health research [36]. National research support organisations may therefore need to target resources and efforts specifically towards supporting AHP team managers, ensuring they have access to relevant training, mentorship and support.

Key research motivators and barriers at an individual level identified by AHPs in our study reflect those reported in almost all previous studies for AHPs and other healthcare professionals in and beyond the UK. These suggest that whilst prioritisation of other job roles and lack of time present common barriers, research is almost universally seen as a positive way for clinical staff to develop skills and derive satisfaction from their jobs. Encouraging research development is likely to lead to more motivated and knowledgeable clinical teams [7, 37]. This is an important consideration for strategic workforce planning in the post-Covid healthcare system where health services are stretched like never before [38] and where reversing the trend of NHS staff leaving service is now viewed as a crucial workstream.

Of further interest is our important finding that research is rarely discussed as a routine part of personal development appraisals among participants in our survey; content analysis from free text responses similarly reflected participants’ perceptions of the low priority given to research activity, the lack of research career pathways, and limited or unclear opportunities for research engagement. The recently published SCORR tool has been developed specifically for clinicians as a tool to aid self-appraisal of research engagement levels. It can be used by individuals or by team-level managers as part of a personal development appraisal to initiate and support research discussions and to inform research development needs. Participants in our survey reported a range of research engagement levels using this tool; the majority rated themselves at level 2 or 3 on the scale, indicating engagement as ‘consumers’ of research evidence to improve clinical care or for service development. These levels meet the expected professional standards for evidence-based clinical practice. The higher levels 4 and 5 on the SCORR scale indicate engagement as ‘producers’ of research evidence, either through supporting research delivery, or through conducting or leading research to generate new evidence. Given the need for AHP-led research, it is encouraging to see a suitable proportion of our survey respondents (21%) rating themselves at these higher levels of research attainment. However, we acknowledge that responder bias may mean that this does not reflect the true situation. Disappointingly, most respondents also reported that they had not engaged in any research-related activity over the previous 12 months. Research capability may not therefore translate readily into research engagement and activity. Survey data from free text responses and from a question about research time allocation (supplementary materials 1, page 6) suggests that this is more likely due to lack of opportunity and time allocated for research rather than lack of aspiration. Using the SCORR tool to support appraisals may help team leaders to identify ‘aspiring researchers’ and ‘research ready’ individuals in their team, and to inform organisation-level discussion around research activity and opportunities.

Mirroring findings from a recent unpublished survey led by CAHPR [39], participants in our survey reported low levels of awareness of research support infrastructures, including CAHPR and NIHR training schemes for AHPs. Whilst declaring clear intentions to support and increase AHP clinical research, these organisations could potentially play a stronger role in promoting and embedding a research culture in healthcare. In particular, there seems to be a need for them to focus attention on support for AHP clinical team managers. This might include ensuring healthcare managers (as well as individual AHP researchers) have access to research mentorship, support networks, information and resources. Closer links with NHS England/NHS Improvement might help to ensure these organisations are visible, relevant and accessible to those working in crucial roles in NHS health and social care to facilitate research engagement.

### Study limitations

The results of this survey should be viewed in the context of several study limitations. Firstly, although the number of participants was significantly greater than any previous studies evaluating research capacity, this still represents only a small proportion of AHPs working in NHS health and social care across the UK. The survey length and ongoing Covid pandemic at the time of survey distribution were potential disincentives for busy clinicians to participate. Based on a random sample of participants, completion times ranging from 9 to 23 min were in line with the estimated completion time of 20 min indicated in the participant information. Despite this potential deterrent, all four UK nations and all 14 AHP professions across a range of healthcare organisations were represented in the survey responses. Furthermore, responses seem to be roughly proportionate with the current balance of professionals in the AHP workforce, reflecting the greater numbers of registered physiotherapist and occupational therapist professionals compared to other professions.

Secondly, with all surveys there is inevitably a risk of self-selection bias towards participants with an interest (and therefore potentially greater engagement) in research, and the proportion of respondents with Masters and PhD level qualifications (49%) and research in their job roles (34%) likely reflects this. It is unclear whether the lower numbers of participants from outside England and from certain professions accurately reflects the proportions of AHPs working in those locations and professions. Alternatively, variation in response rates might be due to different levels of research engagement, or due to other factors such as challenges with the distribution and promotion of the survey. Nevertheless, the number of participants was significantly greater than any previous studies evaluating research capacity and can be considered to provide a fair representation of the views of a wide range of AHPs working across different geographic locations and health and social care settings.

Thirdly, whilst the RCC tool probably represents the best tool currently available for assessing research capacity and capability, it may not be sensitive enough to evaluate all aspects and levels of research capacity. Research capacity frameworks identify factors which might be better evaluated using methods other than self-report questionnaire, such as research partnerships, publications, investment in infrastructure, and planning for sustainability and continuity [20]. Furthermore, a cross-sectional survey provides a snapshot of perceptions at a single time-point and is not able to identify trends over time. The tool does, however, provide a clear insight into current perceptions among AHPs of the research capacity and culture in the NHS at organisation-, team- and individual-level. The study results might therefore be used as a baseline against which to evaluate the future impact of strategic interventions targeting AHP research capacity and culture.

Finally, in this manuscript, we have only presented topline results from our initial analysis of the data generated in this study. Whilst this provides invaluable information that will inform implementation of national research capacity strategies for AHPs, further in-depth analysis will provide an understanding of differences in research capacity and culture between different regions, professions and healthcare organisations that will be of interest to a variety of stakeholders.

Despite these limitations, this first ever national survey provides an important evaluation of the individual challenges, motivators, and confidence levels in research among AHPs. It also highlights where organisation-level research support is sufficient and where it could be improved, and has exposed the team-level inadequacies that need addressing in order to unblock future AHP research potential.

## Conclusions and recommendations

AHPs who responded to this survey indicated that research capacity and culture is adequate at individual and organisational levels, but not at team level. Individuals report feeling motivated to engage in research to develop their skills and increase job satisfaction. However the reality of embedding research into AHP clinical roles and implementing research capacity building strategies at team level poses challenges. Key barriers seem to reflect a lack of prioritisation of research within everyday healthcare, despite recognition of the clear link between research and better outcomes for individuals and the NHS, and acknowledgement that research is the single most important way we can improve our healthcare [10].

Based on the survey responses, and in the context of the HEE research strategy aim of transforming AHP professional identities, culture and roles, we would make the following recommendations:

- at national strategic level: improve visibility of research support organisations, and ensure they are relevant to and provide much needed support targeted at AHP clinical managers to develop a stronger AHP research culture in NHS health and social care teams.
- at organisation-level: ensure that organisations include a focus on AHP research posts and career pathways including clinical academic joint contracts in their research strategies, provide administrative support and software resources, and support middle managers in implementing the research strategy.
- at team-level: introduce routine discussions focusing on research engagement, including during professional development appraisals, and capitalise on the positive benefits from research activity identified by AHPs (development of skills, job satisfaction) that are likely to impact on staff recruitment and retention
- at individual-level: build on existing individual motivation, encourage use of a self-appraisal framework or tool (such as the SCORR tool) to identify research development needs and aspirations that might include generation of new knowledge and implementing research.

The findings from this survey provide a useful baseline against which to measure the impact of future research capacity building initiatives. They also set a national benchmark against which individual professions and healthcare organisations can measure their own research capacity and culture.

## Supplementary Information

**Additional file 1.** AHP Research National Survey Questionnaire.

**Additional file 2.** Results summary report for AHP Research National Survey.

**Additional file 3.** Infographic summary of survey and key findings.

## Data Availability

The datasets generated and analysed during the current study are available in the Leeds Research Data Repository (RADAR), DOI https://doi.org/10.5518/1140. The dataset deposited in the repository excludes data from participants who did not provide consent for public sharing of their survey responses.
