# Supplementary material for: Allied health professionals’ perceptions of research in the United Kingdom national health service: a survey of research capacity and culture
Source: BMC Health Serv Res. 2022 Aug 27;22:1094. doi: 10.1186/s12913-022-08465-6 (PMC9420271; doi:10.1186/s12913-022-08465-6)
Supplement: Supplementary file 1 — Additional file 1. AHP Research National Survey Questionnaire. [file 12913_2022_8465_MOESM1_ESM.docx]

**SUPPLEMENTARY MATERIALS 1 AHP Research National Survey Questionnaire**

# **AHP perceptions of research in the NHS – A national survey**

## Participant Information

**Why are we asking people to complete this Survey?**

This is the first ever national survey to find out how AHPs in the NHS view research. NHS Trusts that support, deliver and implement high quality clinical research are likely to provide better care for patients, to have more informed and motivated staff, offer greater career development opportunities, and benefit economically. Improvements in healthcare depend on evidence generated through clinical research. Allied Health Professionals (AHPs) are increasingly expected to contribute to research activity and are well-placed to lead research that generates evidence for their expanding clinical roles. However, we have scant information about current AHP research activity across the NHS. We also know very little about how confident or how well supported AHPs feel within their teams and organisations to engage in research activity.

**Who can take part in this survey?**

If you are an AHP (see list below) working at least one day per week in the NHS or an organisation that provides NHS-funded healthcare in England, Scotland, Wales or Northern Ireland, we would love to hear from you. It doesn't matter if you are already involved in research, or have no experience of research at all - we would really like you to share your views.

**Who is conducting this survey?**

The survey is led by the AHP Research Champions appointed by the National Institute for Health Research (NIHR) and Council for Allied Health Professions Research (CAHPR). The study has been reviewed by the Health Research Authority IRAS 277676 and is sponsored by Leeds Community Healthcare NHS Trust.

**What will happen to the information I provide in the survey?**

The information you share will be used to provide the first ever national overview of AHPs’ perceptions about their own, their team’s, and their organisation’s ability to engage in different levels of research-related activities. The survey questions also ask about barriers and facilitators, and how confident you feel both in your own abilities, and the support of your organisation in enabling you to be involved in research.  You will only be asked for information about your work and experience that will be used in the study, and you will not be asked to provide any personally identifiable data. We will keep the information you provide safe and secure. Once we have finished the study, we will keep the data so we can check the results. We may use the survey results to compare views in different professions, healthcare organisations and regions.  We will write our reports in a way that no-one can work out that you took part in the study.

We plan to make the anonymised data from this survey widely available according to open science principals so that it can be used by other researchers and can be used to inform future strategies to support AHP research. With your permission, this will include your anonymised data.   You can read more about how data from research participants is used here [www.hra.nhs.uk/patientdataandresearch](http://www.hra.nhs.uk/patientdataandresearch). Alternatively, if you have any queries, complaints or concerns, you can contact: [Thomas.Osborne6@nhs.net](mailto:Thomas.Osborne6@nhs.net), Research Feasibility and Data Co-Ordinator, Leeds Community Healthcare NHS Trust.

**If you would like to take part, please complete all sections of the questionnaire, which should take around 20 minutes**. The survey starts with questions about you, your work, your current research activity/engagement, and awareness of research support services and facilities.  You will then be asked your opinion on:

1. Your own research activity, skills and confidence

2. Support within your team / service for research activity and engagement

3. How well research activity and engagement is supported across your healthcare organisation / Trust

**Remember that we want to hear from everyone, whether you are already involved in research or have no research experience.**

**Thank you for taking the time to complete this survey.  Your responses will be really valuable.**

Chief Investigator, Dr Christine Comer: email c.comer@leeds.ac.uk

Central Study team: Nikki Cullum, Carol Payne, Richard Collings, Alison McCracken

On behalf of the NIHR / CAHPR AHP Research Champions network.

AHP professions invited to participate in this survey

- Art Therapists
- Chiropodist/ Podiatrist
- Dietitian
- Drama therapist
- Hearing aid dispenser
- Music therapist
- Occupational therapist
- Operating department practitioner
- Orthoptist
- Osteopath
- Paramedic
- Physiotherapist
- Practitioner psychologist
- Prosthetist/orthotist
- Radiographer
- Speech and Language therapist
- Clinical scientist
- Biomedical scientist

Before starting the survey, please check that this survey is for you by reading and confirming each of the boxes below.  Please make sure that you have read and understood the participant information. You will then be able to open the survey by selecting the box to indicate that you have understood the information and are happy to complete the questionnaire. Please first confirm that you are a qualified/registered Allied Health Professional (AHP) from the following list: Art therapist, Music therapist, Drama therapist, Biomedical scientist, Chiropodist/ podiatrist, Clinical Scientist, Dietitian, Hearing aid dispenser, Operating department practitioner, Orthoptist, Occupational therapist, Osteopath, Paramedic, Physiotherapist, Prosthetist/ orthotist, Practitioner psychologist, Radiographer, Speech and language therapist. Please confirm that you are currently working in the UK (England, Scotland, Wales or Northern Ireland), either in the NHS or in a healthcare organisation providing NHS-funded healthcare. If you are not an AHP or not currently working in NHS healthcare in the UK, thank you for your interest but this survey is not for you. *Required*

Have you read and understood the participant information?

Are you happy to participate in this survey?

Are you happy for your anonymised responses to be included as part of anonymised data shared with researchers and/or shared to inform future AHP research strategies?

## ABOUT YOU

Where in the UK do you currently work? *Required*

 England

 Scotland

 Wales

 Northern Ireland

 Channel Islands

 Isle of Man

**From the list below, which region of Wales do you work in?**These options are based on the Council for Allied Health Profession Research (CAHPR) hubs across Wales - if you are unsure, please select the CAHPR hub which you think is closest to representing your region

**From the list below, which region of Scotland do you work in?**These options are based on the Council for Allied Health Profession Research (CAHPR) hubs across Scotland - if you are unsure, please select the CAHPR hub which you think is closest to representing your region

**From the list below, which region of England do you work in?** These options are based on the NIHR Clinical Research Networks across England - if you are unsure, please select the response which you think is closest to representing your region

**What is your profession**? *Optional*

 Occupational Therapist

 Physiotherapist

 Radiographer (diagnostic and therapeutic)

 Podiatrist/Chiropodist

 Dietitian

 Speech and language therapist

 Music therapist

 Art therapist

 Drama therapist

 Prosthetist/orthotist

 Paramedic/ Emergency Care Practitioner

 Operating Department Practitioner

 Orthoptist

 Osteopath

 Practitioner Psychologist

 Clinical Scientist

 Biomedical Scientist

 Hearing aid dispenser

 Other

If you selected Other, please specify:

**How long have you been qualified?** *Required*

 0-5 years

 6-10 years

 11-15 years

 16-20 years

 More than 20 years

**Please indicate your highest level of professional / academic qualification** *Optional*

 No formal professional/ academic qualification

 Certificate / Diploma

 Degree

 MSc/ post-graduate

 PhD

 Other

If you selected Other, please specify:

## About Your Work

**What kind of NHS / healthcare organisation do you work in?** (if you have more than one role, please answer using information about your primary AHP role) *Required*

 NHS Acute Trust

 NHS Ambulance Trust

 NHS Community/ Care Trust

 NHS Mental Health Trust

 GP practice

 Primary Care Network

 Clinical Commissioning Group

 Local authority providing NHS-funded health or social care

 Independent provider of NHS-funded healthcare

 Other

If you selected Other, please specify:

**What is your current payband?** If you hold a split role at different pay levels, please indicate your highest payband *Optional*

 Band 2

 Band 3

 Band 4

 Band 5

 Band 6

 Band 7

 Band 8a

 Band 8b

 Band 8c

 Other

If you selected Other, please specify:

**Please state whether research related activities are part of your role description** *Required*

 Yes

 No

**How much of your time in your current role is formally allocated for research or research-related activity?**

 Less than 25% of my time

 more than 25% but less than 50% of my time

 more than 50% but less than 75% of my time

 More than 75% of my time

**Is research engagement/ activity discussed as part of your annual appraisal?**

 Yes routinely

 Only if I bring it up/ or when I am currently involved in research

 No

**Using the scale below which is designed as an appraisal tool for non-medical health professions, please select the response which you think most accurately describes your current level of attainment in clinical research skills.**  If you would like to see the appraisal scale in more detail (with examples), please click on the 'more information' button.

**Are you currently enrolled in any further higher degree study or other professional development related to research?***Optional*

 Yes

 No

**If yes, please indicate what level of study you are enrolled in**

 Undergraduate

 Postgraduate certificate/ diploma

 Postgraduate Masters level

 PhD

**Does your Trust / organisation offer:**

|  | Yes | No | Unsure |
| --- | --- | --- | --- |
| Access to software /statistical packages for research? |  |  |  |
| Library access? |  |  |  |
| Formal research supervision/mentorship for AHPs (eg via academic links?) |  |  |  |
| Informal research supervision/ mentorship? |  |  |  |
| Allocated time provided by Trust/organisation for AHPs to be involved in research? |  |  |  |
| Funding within Trust to support AHP research? |  |  |  |
| Encouragement/ support to apply for external research funding? |  |  |  |
| Administrative support for AHP research activity? |  |  |  |
| Training in research within the Trust/ organisation for AHPs? |  |  |  |
| Support (time/ funding) for AHPs to attend external research training? |  |  |  |
| Support (time/ funding) for AHPs to attend research conferences? |  |  |  |
| Information about what research is happening in the Trust? |  |  |  |
| Opportunities for AHPs to be involved in delivering research? |  |  |  |
| Opportunities for AHPs to be a Principal Investigator? |  |  |  |
| Joint University/ Trust contracts for AHP clinical academics? |  |  |  |
| Does it support / promote AHP clinical academic careers? |  |  |  |

**Please comment on any of the above issues indicating the item you are commenting on.**

**At what level do you currently engage in research? Tick (**⎫**) as many as apply** *Optional*

 I don't currently use/ engage in research at all

 I use research evidence to inform my clinical practice

 I am involved in clinical audit / research activity to evaluate and/ or improve clinical services

 I raise awareness/signpost patients to clinical trials in my area

 I support clinical trials/ research through screening/ recruitment/ treatment delivery

 I act as an expert advisor/ sit on steering groups or research studies

 collaborator/ co-applicant for research studies/ trials

 I take on the role of Site Principal Investigator

 I develop and lead research studies/ trials and/ or act as Chief investigator

 I take part in / run a journal club

 I peer review journal articles/ conference abstracts

 Other

If you selected Other, please specify:

**Please indicate if you have completed any of the following research activities in the past 12 months. Tick (**⎫**) as many as apply**

 Secured research funding

 Co-authored a research-based paper for publication

 Presented research findings at a conference

 No research activity completed in the past 12 months

 Other

If you selected Other, please specify:

**Please indicate any research activity you are currently involved with. Tick (**⎫**) as many as apply**

 Writing a research report, presentation or paper for publication

 Writing a research protocol

 Submitting an ethics application

 Collecting data e.g. surveys, interviews

 Analysing qualitative research data

 Analysing quantitative research data

 Writing a literature review

 Applying for research funding

 Not currently involved with research

 Other

If you selected Other, please specify:

## Awareness of UK research facilities and infrastructure

**Please indicate your level of knowledge or awareness of each of the following national and regional services that support AHP research**by selecting a score on a 1-5 scale where 1=no knowledge or awareness (never heard of them) and 5=in-depth knowledge/ awareness *Optional*

|  | 1 no knowledge/ awareness, never heard of them | 2 heard of them, but little knowledge/ awareness | 3 some knowledge/ awareness | 4 fairly good knowledge/ awareness | 5 in-depth knowledge/ awareness | * Not applicable |
| --- | --- | --- | --- | --- | --- | --- |
| Council for Allied Health Professions Research (CAHPR) |  |  |  |  |  |  |
| National Institute for Health Research (NIHR) in England (*please select 'not applicable if you are not based in England) |  |  |  |  |  |  |
| Integrated Clinical Academic (ICA) Programme (NIHR/Health Education England) (*please answer 'Not applicable' if you are not based in England) |  |  |  |  |  |  |
| The Health and Social Care Public Health Agency (HSC PHA) in Northern Ireland (*please select 'not applicable' if you are not based in Northern Ireland) |  |  |  |  |  |  |
| Health and Social Care Northern Ireland (HSCNI) Fellowship Awards (*please select 'not applicable' if you are not based in Northern Ireland) |  |  |  |  |  |  |
| Northern Ireland Clinical Research Network (NICRN) (*please select 'not applicable' if you are not based in Northern Ireland) |  |  |  |  |  |  |
| The Chief Scientist Office (CSO) in Scotland (*please select 'not applicable' if you are not based in Scotland) |  |  |  |  |  |  |
| NHS Research Scotland (NRS) career researcher fellowships (*please select 'not applicable' if you are not based in Scotland) |  |  |  |  |  |  |
| Health and Care Research Wales (*please select 'not applicable' if you are not based in Wales) |  |  |  |  |  |  |
| Research Capacity Building Collaboration (RCBC) Wales (*please select 'not applicable' if you are not based in Wales) |  |  |  |  |  |  |

**Please comment on any of the above issues indicating the item you are commenting on.**

## YOUR PERCEPTIONS OF YOUR OWN RESEARCH SKILLS AND CONFIDENCE

**Please rate your own current success or skill level for each of the following aspects**by circling a score on a 1-10 scale, where 1=no success/skill (complete novice) and 10=highest possible success/skill (expert)

|  | 1 | 2 | 3 | 4 | 5 | 6 | 7 | 8 | 9 | 10 | Unsure |
| --- | --- | --- | --- | --- | --- | --- | --- | --- | --- | --- | --- |
| Finding relevant literature |  |  |  |  |  |  |  |  |  |  |  |
| Critically reviewing the literature |  |  |  |  |  |  |  |  |  |  |  |
| Using a computer referencing system (e.g. Endnote) |  |  |  |  |  |  |  |  |  |  |  |
| Writing a research protocol |  |  |  |  |  |  |  |  |  |  |  |
| Securing research funding |  |  |  |  |  |  |  |  |  |  |  |
| Submitting an ethics application |  |  |  |  |  |  |  |  |  |  |  |
| Designing questionnaires |  |  |  |  |  |  |  |  |  |  |  |
| Collecting data e.g. surveys, interviews |  |  |  |  |  |  |  |  |  |  |  |
| Using computer data management systems |  |  |  |  |  |  |  |  |  |  |  |
| Analysing qualitative research data |  |  |  |  |  |  |  |  |  |  |  |
| Analysing quantitative research data |  |  |  |  |  |  |  |  |  |  |  |
| Writing a research report |  |  |  |  |  |  |  |  |  |  |  |
| Writing for publication in peer-reviewed journals |  |  |  |  |  |  |  |  |  |  |  |
| Providing advice to less experienced researchers |  |  |  |  |  |  |  |  |  |  |  |

**Please comment on any of the above issues indicating the item you are commenting on.**

**What are the barriers to research for you personally? Tick (**⎫**) as many as apply**

 Lack of time for research

 Lack of suitable backfill

 Other work roles take priority

 Lack of funds for research

 Lack of support from management

 Lack access to equipment for research

 Lack of administrative support

 Lack of software for research

 Isolation

 Lack of library/internet access

 Not interested in research

 Other personal commitments

 Desire for work / life balance

 Lack of a co-ordinated approach to research

 Lack of skills for research

 Intimidated by research language

 Limited by fear of getting it wrong

 Lack of library/internet access

 Lack of diversity and inclusion in research

 Other

If you selected Other, please specify:

**What are the motivators to do research for you personally? Tick (**⎫**) as many as apply**

 To develop skills

 Career advancement

 Increased job satisfaction

 Study or research scholarships available

 Dedicated time for research

 Research written into role description

 Colleagues doing research

 Mentors available to supervise

 Research encouraged by managers

 Grant funds

 Links to universities

 Forms part of Post Graduate study

 Opportunities to participate at own level

 Problem identified that needs changing

 Desire to prove a theory / hunch

 To keep the brain stimulated

 Increased credibility

 Desire to improve sustainability (eg environmental, societal and economic sustainability)

 Other

If you selected Other, please specify:

## YOUR PERCEPTIONS OF YOUR TEAM'S RESEARCH ENGAGEMENT AND SUPPORT

**If you work as part of a team (eg a clinical service, professional team), please rate your team’s success or skill level for each of the following aspects**by circling a score on a 1-10 scale where 1=no success/skill (completely inadequate) and 10=highest possible success/skill (highest level of excellence)

| My Team…. | 1 | 2 | 3 | 4 | 5 | 6 | 7 | 8 | 9 | 10 | Unsure | I do not work as part of a team |
| --- | --- | --- | --- | --- | --- | --- | --- | --- | --- | --- | --- | --- |
| has adequate resources to support staff research training |  |  |  |  |  |  |  |  |  |  |  |  |
| has funds, equipment or admin to support research activities |  |  |  |  |  |  |  |  |  |  |  |  |
| participates in team level planning for research development |  |  |  |  |  |  |  |  |  |  |  |  |
| ensures staff involvement in developing that plan |  |  |  |  |  |  |  |  |  |  |  |  |
| has team leaders that support research |  |  |  |  |  |  |  |  |  |  |  |  |
| provides opportunities to get involved in research |  |  |  |  |  |  |  |  |  |  |  |  |
| undertakes planning that is guided by evidence |  |  |  |  |  |  |  |  |  |  |  |  |
| has consumer involvement in research activities/planning |  |  |  |  |  |  |  |  |  |  |  |  |
| has applied for external funding for research |  |  |  |  |  |  |  |  |  |  |  |  |
| conducts research activities relevant to practice |  |  |  |  |  |  |  |  |  |  |  |  |
| supports applications for research scholarships/ degrees |  |  |  |  |  |  |  |  |  |  |  |  |
| has mechanisms to monitor research quality |  |  |  |  |  |  |  |  |  |  |  |  |
| has identified experts accessible for research advice |  |  |  |  |  |  |  |  |  |  |  |  |
| disseminates research results at research forums/seminars |  |  |  |  |  |  |  |  |  |  |  |  |
| supports a multi-disciplinary approach to research |  |  |  |  |  |  |  |  |  |  |  |  |
| has incentives & support for mentoring activities |  |  |  |  |  |  |  |  |  |  |  |  |
| has external partners (e.g. universities) engaged in research |  |  |  |  |  |  |  |  |  |  |  |  |
| supports peer-reviewed publication of research |  |  |  |  |  |  |  |  |  |  |  |  |
| has software available to support research activities |  |  |  |  |  |  |  |  |  |  |  |  |

**Please comment on any of the above issues indicating the item you are commenting on.**

**What are the biggest barriers to research in your team?**

**What are the biggest motivators to research in your team?**

## YOUR PERCEPTIONS OF RESEARCH SUPPORT & CAPACITY WITHIN THE TRUST/ORGANISATION YOU WORK FOR

**Please indicate how supported you feel to engage in research in your workplace by rating your organisation’s success or skill level for each of the following aspects**by circling a score on a 1-10 scale where 1=no success/skill (completely inadequate) and 10=highest possible success/skill (highest level of excellence)

| My Trust/ organisation… | 1 | 2 | 3 | 4 | 5 | 6 | 7 | 8 | 9 | 10 | Unsure |
| --- | --- | --- | --- | --- | --- | --- | --- | --- | --- | --- | --- |
| has adequate resources to support staff research training |  |  |  |  |  |  |  |  |  |  |  |
| has funds, equipment or admin to support research activities |  |  |  |  |  |  |  |  |  |  |  |
| has a plan or policy for research development |  |  |  |  |  |  |  |  |  |  |  |
| has senior managers that support research |  |  |  |  |  |  |  |  |  |  |  |
| ensures staff career pathways are available in research |  |  |  |  |  |  |  |  |  |  |  |
| ensures organisation planning is guided by evidence |  |  |  |  |  |  |  |  |  |  |  |
| has consumers involved in research |  |  |  |  |  |  |  |  |  |  |  |
| accesses external funding for research |  |  |  |  |  |  |  |  |  |  |  |
| promotes clinical practice based on evidence |  |  |  |  |  |  |  |  |  |  |  |
| encourages research activities relevant to practice |  |  |  |  |  |  |  |  |  |  |  |
| has software programs for analysing research data |  |  |  |  |  |  |  |  |  |  |  |
| has mechanisms to monitor research quality |  |  |  |  |  |  |  |  |  |  |  |
| has identified experts accessible for research advice |  |  |  |  |  |  |  |  |  |  |  |
| supports a multi-disciplinary approach to research |  |  |  |  |  |  |  |  |  |  |  |
| has regular forums/bulletins to present research findings |  |  |  |  |  |  |  |  |  |  |  |
| engages external partners (e.g. universities) in research |  |  |  |  |  |  |  |  |  |  |  |
| supports applications for research scholarships/ degrees |  |  |  |  |  |  |  |  |  |  |  |
| supports the peer-reviewed publication of research |  |  |  |  |  |  |  |  |  |  |  |

**Please comment on any of the above issues indicating the item you are commenting on.**

## Questions to help us understand diversity within research. These questions are optional

Ethnicity

 Asian

 Black

 White

 Asian British - Bangladeshi

 Asian British - Indian

 Asian British - Pakistani

 Asian - Chinese

 Asian - Other

 Black British - African

 Black British - Caribbean

 Black British - Other

 Mixed - Black African and White

 Mixed - Black Asian and White

 Mixed - Caribbean and White

 Mixed - Other

 White - British

 White - Irish

 White - Irish/Romany

 White - Other

 Other

Gender

 female

 male

 prefer not to state

 non-binary

 other

Sexual orientation

 bisexual

 gay

 lesbian

 heterosexual (straight)

 prefer not to state

 other

Disability

 none

 physical

 learning

 hidden

 prefer not to state

**THANK YOU VERY MUCH FOR TAKING THE TIME TO COMPLETE THIS QUESTIONNAIRE**

**Your responses are greatly valued. The information you have provided will be used to identify ways in which AHP research activity and engagement can be better supported, and may be used for further research and evaluation. All responses are and will remain anonymous.**

This survey is based on the Research Capacity in Context questionnaire, developed by Queensland Health and Griffith University; Sue Pager, Metro South Hospital and Health Service, Brisbane, QLD  Susan_pager@health.qld.gov.au  [The document is licensed under Creative Commons [*http://creative*](http://creative/)commons.org/licenses/by/2.5/au/]

Within this survey, questions are incorporated from the 'Clinicians' Skills, Capability, and Organisational Research Readiness (SCORR) tool.  [Iles-Smith, H., Burnett, C., Ross, D.H. and Siddle, H.J., 2019.  International Journal of Practice-Based Learning in Health and Social Care, 7(2), pp.57-68]
