# Supplementary material for: Allied health professionals’ perceptions of research in the United Kingdom national health service: a survey of research capacity and culture
Source: BMC Health Serv Res. 2022 Aug 27;22:1094. doi: 10.1186/s12913-022-08465-6 (PMC9420271; doi:10.1186/s12913-022-08465-6)
Supplement: Supplementary file 2 — Additional file 2. Results summary report for AHP Research National Survey. [file 12913_2022_8465_MOESM2_ESM.docx]

**SUPPLEMENTARY MATERIALS 2 Results summary report for AHP Research National Survey**

*This summary report includes anonymous data from all study participants. Data from 3276 participants who provided permission will be uploaded to a public online research data repository. This data can be accessed upon reasonable request to inform further research and strategies.*

**Q1 Eligibility and consent questions**

| **Eligibility and consent** | **Number** | **Percentage** |
| --- | --- | --- |
| I am a qualified Allied Health Professional (see AHP list above) working in the NHS, local authority, or organisation providing NHS-funded healthcare in the UK | 3344 | 100 |
| I have read and understood the participant information | 3344 | 100 |
| I am happy to take part in the survey by completing the questionnaire | 3344 | 100 |
| I am happy for my anonymised data to be shared with researchers and/or shared to inform future AHP research strategies? | 3276 | 98 |

**DEMOGRAPHIC DATA**

**Q2-7 Demographic questions**

(Profession, length of time qualified, pay band, type of healthcare organisation, country/region questions added by Champions

Highest level of qualification RCC question, but with modified response options)

| Profession | **Number** | **Percentage** |
| --- | --- | --- |
| Occupational Therapist | 747 | 22.4 |
| Physiotherapist | 1134 | 33.9 |
| Radiographer (diagnostic and therapeutic) | 240 | 7.2 |
| Podiatrist/Chiropodist | 160 | 4.8 |
| Dietitian | 268 | 8.0 |
| Speech and language therapist | 328 | 9.8 |
| Music therapist | 17 | 0.5 |
| Art therapist | 25 | 0.7 |
| Drama therapist | 7 | 0.2 |
| Prosthetist/orthotist | 33 | 1.0 |
| Paramedic/ Emergency Care Practitioner | 70 | 2.1 |
| Operating Department Practitioner | 62 | 1.9 |
| Orthoptist | 49 | 1.5 |
| Osteopath | 5 | 0.1 |
| Practitioner Psychologist* | 54 | 1.6 |
| Clinical Scientist* | 36 | 1.1 |
| Biomedical Scientist* | 36 | 1.1 |
| Hearing aid dispenser* | 1 | >0.1 |
| Other** | 69 | 2.1 |
| ** professions in shaded boxes are not on the NHS England list of 14 Allied Health Professions, but are health professions council registered professions*  ***Despite all participants confirming that they are a qualified AHP, 69 participants identified themselves as ‘other’ professions; these included 12 nurses, 7 sonographers, 6 pharmacists, 5 social workers, 5 participants in research/trial co-ordination or governance roles, and 34 others including 2 GPs.* | | |

| Length of time qualified | **AHPs**  **n (%)** | **All participants n (%)** |
| --- | --- | --- |
| 0-5 years | 525 (16.7) | 571 (17.1) |
| 6-10 years | 472 (15.0) | 500 (15.0) |
| 11-15 years | 533 (16.9) | 567 (17.0) |
| 16-20 years | 484 (15.4) | 516 (15.4) |
| More than 20 years | 1131 (36%) | 1190 (35.6) |
| Highest level of qualification |  |  |
| No formal professional/ academic qualification | 1 (<0.1) | 5 (0.1) |
| Certificate / Diploma | 173 (5.5) | 181 (5.4) |
| Degree | 1418 (45.1) | 1454 (43.5) |
| MSc/ post-graduate | 1363 (43.4.) | 1451 (43.4) |
| PhD | 166 (5.3) | 214 (6.4) |
| Other | 21 (0.7) | 35 (1.0) |
| **Current pay band** |  |  |
| Band 2 | 0 | 1(>0.1) |
| Band 3 | 0 | 3 (0.1) |
| Band 4 | 2 (0.1) | 7 (0.2) |
| Band 5 | 242 (7.7) | 253 (7.6) |
| Band 6 | 1011 (32.2) | 1042 (31.3) |
| Band 7 | 1180 (37.6) | 1242 (37.3) |
| Band 8a | 474 (15.1) | 521 (15.6) |
| Band 8b | 132 (4.2) | 149 (4.5) |
| Band 8c | 50 (1.6) | 56 (1.7) |
| Other | 45 (1.4) | 59 (1.8) |
| Type of Healthcare Organisation/ Trust |  |  |
| NHS Acute Trust | 1659 (52.8) | 1760 (52.6) |
| NHS Ambulance Trust | 43 (1.4) | 43 (1.3) |
| NHS Community/ Care Trust | 900 (28.6) | 916 (27.4) |
| NHS Mental Health Trust | 306 (9.7) | 357 (10.7) |
| GP practice | 16 (0.5) | 25 (0.7) |
| Primary Care Network | 42 (1.3) | 45 (1.3) |
| Clinical Commissioning Group | 8 (0.3) | 8 (0.2) |
| Local authority providing NHS-funded health or social care | 30 (1) | 34 (1.0) |
| Independent provider of NHS-funded healthcare | 36 (1.1) | 40 (1.2) |
| Other | 105 (3.3) | 116 (3.5) |

| Country & Region | **Region** | **AHPs**  **n (%)** | **All participant n (%)** |
| --- | --- | --- | --- |
| England | North East and North Cumbria | 163 (5.6) | 167 (5.4) |
|  | North West Coast | 208 (7.1) | 216 (6.9) |
|  | Yorkshire and Humber | 351 (12.0) | 377 (12.1) |
|  | Greater Manchester | 148 (5.1) | 166 (5.3) |
|  | East Midlands | 266 (9.1) | 285 (9.1) |
|  | West Midlands | 243 (8.3) | 250 (8.0) |
|  | West of England | 119 (4.1) | 130 (4.2) |
|  | Thames Valley and South Midlands | 107 (3.7) | 112 (3.6) |
|  | Eastern | 273 (9.3) | 287 (9.2) |
|  | Kent, Surrey and Sussex | 220 (7.5) | 234 (7.5) |
|  | Wessex | 103 (3.5) | 108 (3.5) |
|  | South West Peninsula | 256 (8.8) | 277 (8.9) |
|  | North Thames | 90 (3.1) | 93 (3.0) |
|  | South London | 171 (5.9) | 196 (6.3) |
|  | North West London | 162 (5.5) | 169 (5.4) |
|  | not sure | 42 (1.4) | 48 (1.5) |
|  | **Total from England** | 2922 (92.9) | 3115 (93.2) |
| Scotland | North of Scotland | 27 (22.3) | 27 (22.0) |
|  | South East Scotland | 25 (20.7) | 25 (20.3) |
|  | West of Scotland | 67 (55.4) | 68 (55.3) |
|  | not sure | 2 (1.7) | 3 (2.4) |
|  | **Total from Scotland** | 121 (3.8) | 123 (3.7) |
| Wales | North Wales | 6 (17.1) | 6 (15.4) |
|  | South, Mid and West Wales | 29 (82.9) | 33 (84.6) |
|  | not sure | 0 | 0 |
|  | **Total from Wales** | 35 (1.1) | 39 (1.2) |
| Northern Ireland | **Total from Northern Ireland** | 67 (2.1) | 67 (2.0) |
| Channel Islands & Isle of Man | **Total from Channel Islands/ Isle of Man** | 0 | 0 |

### *Percentages in shaded boxes represent percentage of survey respondents from each country.*

*Percentages in unshaded boxes represent percentage responding from each region within that country*

**Q24- 27 Diversity Questions** (in line with CAHPR Review survey)

| Ethnicity | **AHPs**  **n (%)** | **All participants n (%)** |
| --- | --- | --- |
| Asian | 18 (0.6) | 18 (0.5) |
| Black | 7 (0.2) | 9 (0.3) |
| White | 1544 (50%) | 1617(49.3) |
| Asian British - Bangladeshi | 2 (0.1) | 5 (0.2) |
| Asian British - Indian | 54 (1.8) | 57 (1.7) |
| Asian British - Pakistani | 10 (0.3) | 11 (0.3) |
| Asian - Chinese | 16 (0.5) | 17 (0.5) |
| Asian - Other | 11 (0.4) | 15 (0.5) |
| Black British - African | 17 (0.6) | 23 (0.7) |
| Black British - Caribbean | 11 (0.4) | 12 (0.4) |
| Black British - Other | 2 (0.1) | 2 (0.1) |
| Mixed - Black African and White | 2 (0.1) | 2 (0.1) |
| Mixed - Black Asian and White | 5 (0.2) | 5 (0.2) |
| Mixed - Caribbean and White | 8 (0.3) | 9 (0.3) |
| Mixed - Other | 29 (0.9) | 32 (1.0) |
| White - British | 1048 (34) | 1121(34.2) |
| White - Irish | 101 (3.3) | 103 (3.1) |
| White - Irish/Romany | 0 | 0 |
| White - Other | 168 (5.4) | 185 (5.6) |
| Other | 32 (1) | 36 (1.1) |
| Gender |  |  |
| female | 2570 (83.8) | 2717(83.3) |
| male | 433 (14.1) | 477(14.6) |
| prefer not to state | 49 (1.6) | 51 (1.6) |
| non-binary | 11 (0.4) | 11 (0.3) |
| other | 4 (0.1) | 4 (0.1) |
| Sexual Orientation |  |  |
| bisexual | 79 (2.6) | 84 (2.6) |
| gay | 41 (1.3) | 46 (1.4) |
| lesbian | 43 (1.4) | 44 (1.3) |
| heterosexual (straight) | 2702(87.8) | 2877(87.9) |
| prefer not to state | 194 (6.3) | 202 (6.2) |
| other | 18 (0.6) | 19 (0.6) |
| Disability |  |  |
| none | 2677 (87.0) | 2844(87.0) |
| physical | 57 (1.9) | 61 (1.9) |
| learning | 79 (2.6) | 83 (2.5) |
| hidden | 159 (5.2) | 173 (5.3) |
| prefer not to state | 104 (3.4) | 109 (3.3) |

**RESEARCH ACTIVITY/ENGAGEMENT, CULTURE AND CAPACITY DATA**

### **Q8(a) Are research-related activities part of your role description?** (RCC question)

| **Research in role description** | **AHPs n (%)** | **All participants n (%)** |
| --- | --- | --- |
| Yes | 1071 (34.1) | 1167 (34.9) |
| No | 2074 (65.9) | 2177 (65.1) |

**Q8(b) If previous answer ‘yes’: How much of your time in your current role is formally allocated for research or research-related activity?** (added by Champions)

| **Time allocated for research/ research-related activity** | **AHPs (1071 responded ‘yes’ for Q8a) n (%)** | **All participants (1167 responded ‘yes’ for Q8a) n (%)** |
| --- | --- | --- |
| Less than 25% of my time | 844 (78.9) | 914 (27.3) |
| >25% but < 50% | 71 (6.6) | 77 (2.3) |
| >50% but less than 75% | 48 (4.5) | 51 (1.5) |
| More than 75% of my time | 107 (10%) | 123 (3.7) |
| missing | 1 (0.1) | 2 (0.2) |

**Q9 Is research engagement/ activity discussed as part of your annual appraisal?** (added by Champions)

| **Discussion of research at appraisal** | **AHPs n (%)** | **All participants n (%)** |
| --- | --- | --- |
| Yes routinely | 560 (17.9) | 610 (18.2) |
| Only if I bring it up/ or when I am currently involved in research | 1557 (49.7) | 1643 (49.1) |
| No | 1017 (32.5) | 1080 (32.2) |
| missing | 11 (0.3) | 11 (0.3) |

**Q10 Current Level of research skill** (SCORR appraisal tool): Please select the response which you think most accurately describes your current level of attainment in clinical research skills


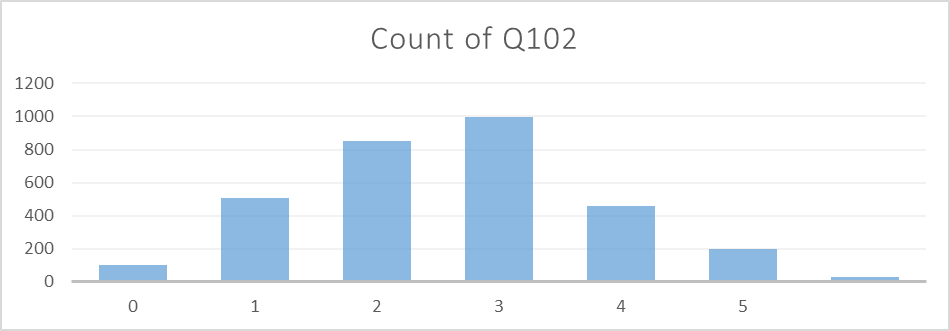


level 0 level 1 level 2 level 3 level 4 level 5

3.3% 16.4% 27.3% 32.0% 14.7% 6.4%

N = 3113

Missing = 32

Mean (SD) = 2.6 (1.2)

Median (IQR) = 3 (2,3)

| **SCORR self-appraisal of research skill level** | **AHPs** | **All participants** |
| --- | --- | --- |
| Level 0 Does not meet Levels 1-5 and requires support to gain knowledge from evidence based on practice/ research and apply it to practice | 103 (3.3) | 121 (3.7) |
| Level 1 Gains new knowledge from evidence/ research and applies it to practice | 509 (16.4) | 544 (16.4) |
| Level 2 Shares awareness of new knowledge (from EBP/research) with colleagues, patients and the public and challenges practice to improve patient care | 849 (27.3) | 888 (26.8) |
| Level 3 Uses research findings to support change and service development to address clinical challenges (eg contributes to established clinical networks, journal clubs, literature reviews, development of local/national policies) | 995 (32.0) | 1040 (31.4) |
| Level 4 Actively undertakes own research with the support of others or supports the delivery of research and disseminates research outcomes locally | 459 (14.7) | 505 (15.2) |
| Level 5 Leads the generation of new knowledge through research (e.g. actively develops and leads clinical research, engages with research collaborators, obtain research funding, disseminates research nationally/internationally) | 198 (6.4) | 214 (6.5) |
| Median (IQR) | 3 (2,3) | 3 (2,3) |
| Mean (SD) | 2.58 (1.20) | 2.55 (1.23) |
| Missing | 32 | 32 |

**Q11(a) Are you currently enrolled in any further higher degree study or other professional development related to research?** (RCC Question)

| **Enrolled in training for research?** | **AHPs**  **n (%)** | **All participants**  **n (%)** |
| --- | --- | --- |
| Yes | 429 (13.7) | 455 (13.6) |
| No | 2692 (86.3) | 2864 (85.6) |
| missing | 24 (0.8) | 25 (0.7) |

**Q11(b) If yes, please indicate what level of study you are enrolled in** (RCC Question with modified response options)

| **Level of research training/ development enrolled in** | **AHPs (429 who responded ‘yes’ to Q11a) n (%)** | **All participants (455 who responded ‘yes’ to Q11a) n (%)** |
| --- | --- | --- |
| Undergraduate | 15 (3.5) | 15 (0.4) |
| Postgraduate certificate/diploma | 65 (15.2) | 69 (2.1) |
| Postgraduate Masters level | 245 (57.1) | 254 (7.6) |
| PhD | 104 (24.2) | 117 (3.5) |
| missing | 0 | 0 |

**Q13, 14, 15. Recent and current research activities and engagement** (Research engagement question added by Champions. Research activity current and in last 12 months are RCC questions)

| At what level do you currently engage in research? | **AHPs**  **n (%)** | **All participants**  **n (%)** |
| --- | --- | --- |
| I don’t currently use/ engage in research at all | 227 (7.2) | 259 (7.7) |
| I use research evidence to inform my clinical practice | 2657 (84.6) | 2795 (83.6) |
| I am involved in clinical audit/ research activity to evaluate and/ or improve clinical services | 2005 (63.9) | 2120 (63.4) |
| I raise awareness/ signpost patients to clinical trials in my area | 752 (24.0) | 803 (24.0) |
| I support clinical trials/ research through screening/ recruitment/ treatment delivery | 656 (20.9) | 706 (21.1) |
| I act as an expert advisor/ sit on steering groups or research studies | 286 (9.1) | 300 (9.0) |
| Collaborator/ co-applicant for research studies/ trials | 344 (11.0) | 370 (11.1) |
| I take on the role of Site Principal Investigator | 239 (7.6) | 249 (7.4) |
| I develop and lead research studies/ trials and/ or act as Chief Investigator | 207 (6.6) | 223 (6.7) |
| I take part in/ run a journal club | 872 (27.8) | 906 (27.1) |
| I peer review journal articles/ conference abstracts | 600 (19.1) | 631 (18.9) |
| Other | 123 (3.9) | 130 (3.9) |
| Research activities undertaken in the past 12 months |  |  |
| Secured research funding | 215 (7.2) | 229 (6.8) |
| Co-authored a research-based paper for publication | 453 (15.2) | 492 (14.7) |
| Presented research findings at a conference | 333 (11.2) | 363 (10.9) |
| No research activity completed in the past 12 months | 2025 (68.1) | 2133 (63.8) |
| Other | 398 (13.4) | 428 (12.8) |
| Current research activities |  |  |
| Writing a research report, presentation or paper for publication | 492 (16.1) | 535 (16.0) |
| Writing a research protocol | 292 (9.6) | 311 (9.3) |
| Submitting an ethics application | 213 (7.0) | 228 (6.8) |
| Collecting data e.g. surveys, interviews | 764 (25.0) | 828 (24.8) |
| Analysing qualitative research data | 306 (10.0) | 332 (9.9) |
| Analysing quantitative research data | 355 (11.0) | 374 (11.2) |
| Writing a literature review | 293 (9.6) | 317 (9.5) |
| Applying for research funding | 256 (8.4) | 271 (8.1) |
| Not currently involved with research | 1803 (59.0) | 1885 (56.4) |
| Other | 166 (5.4) | 182 (5.4) |

**Q16. Awareness of research support infrastructure / organisations** (added by research Champions)

|  | No knowledge/ never heard of them | Heard of them but little knowledge / awareness | Some knowledge/ awareness | Fairly good knowledge/ awareness | In-depth knowledge/ awareness | Not applicable |
| --- | --- | --- | --- | --- | --- | --- |
| Council for Allied Health Professions Research (CAHPR) (3344 all participants across all countries)* | 1097 (33) | 1063 (32) | 693 (21) | 355 (10) | 121 (4) | 4 (0) |
| Council for Allied Health Professions Research (CAHPR) (3145 AHPs across all countries)** | 983 (31) | 1017 (33) | 669 (21) | 347 (11) | 116 (4) | 4 (0) |
| NIHR England (2922 AHPs in England | 281 (10) | 781 (27) | 931 (32) | 656 (22) | 260 (9) | 7 (0) |
| Integrated Clinical Academic (ICA) training programmes NIHR/HEE (2922 AHPs in England) | 1557 (54) | 577 (20) | 324 (11) | 217 (7) | 192 (7) | 43 (1) |
| The Health and Social Care Public Health Agency (HSC PHA) in Northern Ireland (67 AHPs in Northern Ireland)) | 7 (11) | 16 (25) | 19 (29) | 17 (26) | 4 (6) | 2 (3) |
| Health and Social Care Northern Ireland (HSCNI) Fellowship Awards (67 AHPs in N.Ireland)) | 27 (40) | 17 (25) | 11 (17) | 9 (13) | 3 (5) | 0 (0) |
| Northern Ireland Clinical Research Network (NICRN) (67 AHPs in N Ireland) | 24 (36) | 17 (26) | 10 (15) | 11 (17) | 1 (1) | 3 (5) |
| The Chief Scientist Office (CSO) in Scotland (121 AHPs in Scotland) | 50 (42) | 29 (24) | 28 (24) | 6 (5) | 5 (4) | 1 (1) |
| NHS Research Scotland (NRS) career researcher fellowships (121 AHPs in Scotland) | 36 (30) | 41 (34) | 28 (23) | 6 (5) | 9 (7) | 1 (1) |
| Health and Care Research Wales (35 AHPs in Wales) | 10 (29) | 10 (29) | 8 (23) | 2 (5) | 5 (14) | 0 (0) |
| Research Capacity Building Collaboration (RCBC) Wales (35 AHPs in Wales) | 21 (60) | 3 (9) | 1 (2) | 3 (9) | 7 (20) | 0(0) |

**Numbers selecting response from all survey participants followed by percentage in parentheses*

*** Numbers selecting response from all participants from 14 NHSE AHP professions (percentage in parentheses)*

*All other values represent response numbers from AHP participants from the relevant country (percentage in parentheses)*

**Q12. Organisation level research opportunities and provision of support** (RCC question with modified response options)

| **Does your Trust / organisation offer:** | **AHPs n (%)** | **All n (%)** |
| --- | --- | --- |
| Access to software /statistical packages for research |  |  |
| Yes | 649 (20.6) | 708 (21.2) |
| No | 604 (19.2) | 648 (19.4) |
| Unsure | 1892 (60.2) | 1988 (59.4) |
| Library Access |  |  |
| Yes | 2836 (90.2) | 3003 (89.8) |
| No | 127 (4.0) | 146 (4.4) |
| Unsure | 182 (5.8) | 195 (5.8) |
| Formal research supervision/mentorship for AHPs (eg via academic links) |  |  |
| Yes | 756 (24.0) | 794 (23.7) |
| No | 844 (26.8) | 903 (27.0) |
| Unsure | 1545 (49.1) | 1647 (49.3) |
| Informal research supervision/ mentorship |  |  |
| Yes | 1336 (42.5) | 1407 (42.1) |
| No | 596 (19.0) | 646 (19.3) |
| Unsure | 1213 (38.6) | 1291 (38.6) |
| Allocated time provided by Trust/organisation for AHPs to be involved in research |  |  |
| Yes | 719 (22.9) | 765 (22.9) |
| No | 1238 (39.4) | 1326 (39.7) |
| Unsure | 1188 (37.8) | 1253 (37.5) |
| Funding within Trust to support AHP research |  |  |
| Yes | 767 (24.4) | 819 (24.5) |
| No | 822 (26.1) | 875 (26.2) |
| Unsure | 1556 (49.5) | 1650 (49.3) |
| Encouragement/ support to apply for external research funding |  |  |
| Yes | 1312 (41.7) | 1377 (41.2) |
| No | 730 (23.2) | 792 (23.7) |
| Unsure | 1103 (35.1) | 1175 (35.1) |
| Administrative support for AHP research activity |  |  |
| Yes | 294 (9.3) | 326 (9.7) |
| No | 1382 (43.9) | 1465 (43.8) |
| Unsure | 1469 (46.7) | 1553 (46.4) |
| Training in research within the Trust/ organisation for AHPs |  |  |
| Yes | 875 (27.8) | 923 (27.6) |
| No | 986 (31.4) | 1047 (31.3) |
| Unsure | 1284 (40.8) | 1374 (41.1) |
| Support (time/ funding) for AHPs to attend external research training |  |  |
| Yes | 1262 (40.1) | 1320 (39.5) |
| No | 668 (21.2) | 728 (21.8) |
| Unsure | 1215 (38.6) | 1296 (38.8) |
| Support (time/ funding) for AHPs to attend research conferences |  |  |
| Yes | 1417 (45.1) | 1484 (44.4) |
| No | 631 (20.1) | 693 (20.7) |
| Unsure | 1097 (34.9) | 1167 (34.9) |
| Information about what research is happening in the Trust |  |  |
| Yes | 1741 (55.4) | 1857 (55.5) |
| No | 694 (22.1) | 735 (22.0) |
| Unsure | 710 (22.6) | 752 (22.5) |
| Opportunities for AHPs to be involved in delivering research |  |  |
| Yes | 1394 (44.3) | 1473 (44.0) |
| No | 641 (20.4) | 688 (20.6) |
| Unsure | 1110 (35.3) | 1183 (35.4) |
| Opportunities for AHPs to be a Principal Investigator |  |  |
| Yes | 705 (22.4) | 746 (22.3) |
| No | 777 (24.7) | 832 (24.9) |
| Unsure | 1663 (52.9) | 1766 (52.8) |
| Joint University/ Trust contracts for AHP clinical academics |  |  |
| Yes | 602 (19.1) | 646 (19.3) |
| No | 874 (27.8) | 926 (27.7) |
| Unsure | 1669 (53.1) | 1772 (53.0) |
| Does it support / promote AHP clinical academic careers |  |  |
| Yes | 884 (28.1) | 925 (27.7) |
| No | 900 (28.6) | 966 (28.9) |
| Unsure | 1361 (43.3) | 1453 (43.5) |

**
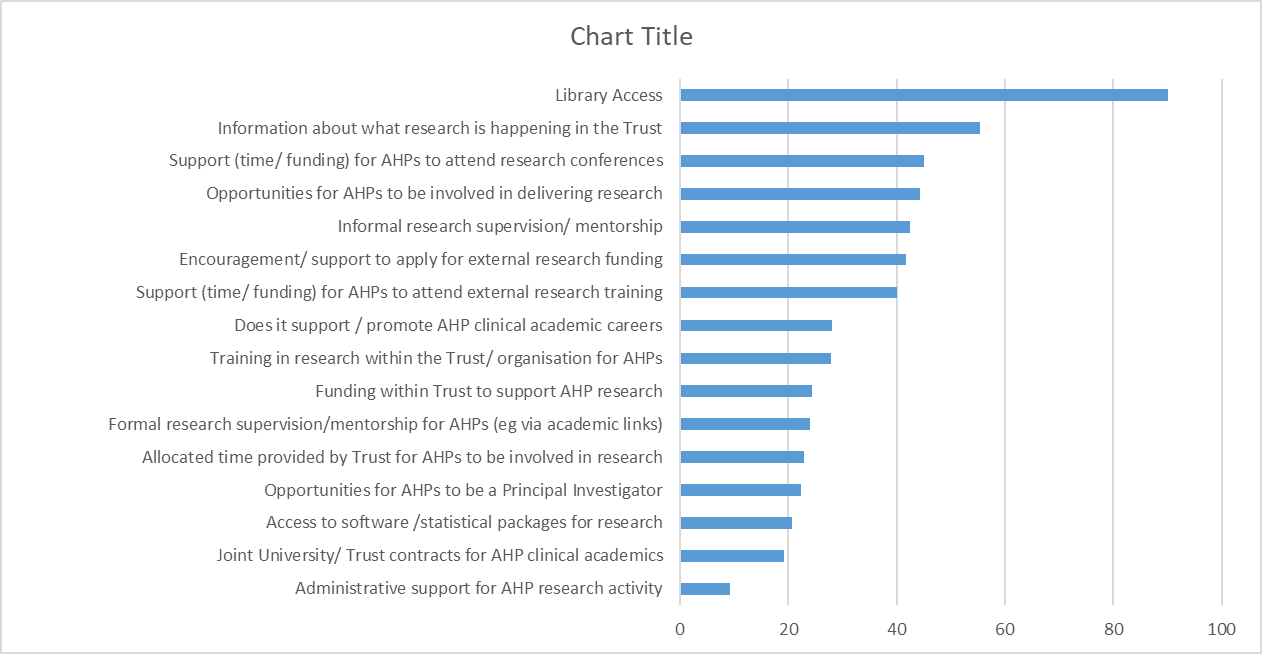
**

**Q17, 20, 23 Research skills/success at organisation level, team level and individual level** (RCC questions)

|  | Median(IQR) | Unsure (%) | Median(IQR) | Unsure (%) |
| --- | --- | --- | --- | --- |
| **Individual level** | AHPs (3145) | | All (3344) | |
| Finding relevant literature | 7 (5-8) | 0.2 | 7 (5-8) | 0.2 |
| Critically reviewing the literature | 7 (5-8) | 0.3 | 7 (5-8) | 0.4 |
| Using a computer referencing system (e.g. Endnote) | 5 (2-7) | 1.8 | 5 (2-7) | 1.9 |
| Writing a research protocol | 3 (2-6) | 1.0 | 4 (2-6) | 1.2 |
| Securing research funding | 1 (1-3) | 2.4 | 1 (1-3) | 2.6 |
| Submitting an ethics application | 2 (1-5) | 1.7 | 2 (1-5) | 1.8 |
| Designing questionnaires | 4 (2-7) | 1.0 | 5 (2-7) | 1.2 |
| Collecting data e.g. surveys, interviews | 5 (3-7) | 0.9 | 5 (3-7) | 1.1 |
| Using computer data management systems | 3 (1-6) | 1.7 | 3 (1-6) | 1.8 |
| Analysing qualitative research data | 4 (2-6) | 0.9 | 4 (2-6) | 1.1 |
| Analysing quantitative research data | 4 (2-6) | 0.9 | 4 (2-6) | 1.1 |
| Writing a research report | 4 (2-7) | 1.1 | 4 (2-7) | 1.2 |
| Writing for publication in peer-reviewed journals | 2 (1-5) | 1.7 | 3 (1-6) | 1.9 |
| Providing advice to less experienced researchers | 2 (1-5) | 1.3 | 2 (1-5) | 1.5 |
| Individual level research skills/success overall score | **4 (2-6)** | **1.2** | **4 (2-6)** | **1.4** |
| **Team level** (2-5% reported they do not work in a team) | AHPs (3145) | | All (3344) | |
| has adequate resources to support staff research training | 3 (1-5) | 9.7 | 3 (1-5) | 9.7 |
| has funds/equipment/admin to support research activities | 2 (1-4) | 11.0 | 2 (1-4) | 10.9 |
| participates in team level planning for research development | 2 (1-4) | 8.5 | 2 (1-4) | 8.6 |
| ensures staff involvement in developing that plan | 2 (1-5) | 9.2 | 2 (1-5) | 9.2 |
| has team leaders that support research | 4 (2-7) | 7.2 | 4 (2-7) | 7.2 |
| provides opportunities to get involved in research | 3 (1-5) | 6.6 | 3 (1-5) | 6.7 |
| undertakes planning that is guided by evidence | 4 (2-7) | 8.8 | 4 (2-7) | 8.8 |
| has consumer involvement in research activities/planning | 2 (1-5) | 12.8 | 2 (1-5) | 12.8 |
| has applied for external funding for research | 1 (1-4) | 14.5 | 1 (1-4) | 14.5 |
| conducts research activities relevant to practice | 3 (1-6) | 9.4 | 3 (1-6) | 9.4 |
| supports applications for research scholarships/ degrees | 2 (1-6) | 13.7 | 2 (1-6) | 13.5 |
| has mechanisms to monitor research quality | 2 (1-5) | 15.6 | 2 (1-5) | 15.6 |
| has identified experts accessible for research advice | 2 (1-6) | 13.2 | 2 (1-6) | 13.3 |
| disseminates research results at research forums/seminars | 2 (1-6) | 11.0 | 2 (1-6) | 10.9 |
| supports a multi-disciplinary approach to research | 3 (1-6) | 10.8 | 3 (1-6) | 10.9 |
| has incentives & support for mentoring activities | 2 (1-4) | 13.8 | 2 (1-4) | 13.8 |
| has external partners (e.g. universities) engaged in research | 3 (1-6) | 12.5 | 3 (1-6) | 12.4 |
| supports peer-reviewed publication of research | 2 (1-6) | 13.6 | 2 (1-6) | 13.5 |
| has software available to support research activities | 1 (1-4) | 18.1 | 1 (1-4) | 18.0 |
| Team level research skills/ success overall score | **2 (1-5)** | **11.6** | **2 (1-5)** | **11.6** |
| **Organisation level** | AHPs (3145) | | All (3344) | |
| has adequate resources to support staff research training | 3 (2-6) | 19.4 | 3 (2-6) | 19.3 |
| has funds/equipment/admin to support research activities | 3 (1-5) | 22.5 | 3 (1-5) | 22.3 |
| has a plan or policy for research development | 4 (2-7) | 24.2 | 4 (2-7) | 24.2 |
| has senior managers that support research | 4 (2-7) | 16.3 | 4 (2-7) | 16.5 |
| ensures staff career pathways are available in research | 2 (1-5) | 21.2 | 2 (1-5) | 21.4 |
| ensures organisation planning is guided by evidence | 4 (2-7) | 19.9 | 4 (2-7) | 20.1 |
| has consumers involved in research | 3 (1-6) | 27.8 | 3 (1-6.25) | 27.8 |
| accesses external funding for research | 4 (1-7) | 27.0 | 4 (1-7) | 27.1 |
| promotes clinical practice based on evidence | 6 (3-9) | 11.3 | 6 (3-9) | 11.5 |
| encourages research activities relevant to practice | 4 (2-7) | 15.2 | 5 (2-7) | 15.2 |
| has software programs for analysing research data | 2 (1-5) | 37.6 | 2 (1-5) | 37.2 |
| has mechanisms to monitor research quality | 3 (1-6) | 33.8 | 3 (1-6) | 33.6 |
| has identified experts accessible for research advice | 4 (1-7) | 26.5 | 4 (1-7) | 26.6 |
| supports a multi-disciplinary approach to research | 4 (1-7) | 22.5 | 4 (1-7) | 22.5 |
| has regular forums/bulletins to present research findings | 3 (1-7) | 20.2 | 3 (1-7) | 20.1 |
| engages external partners (e.g. universities) in research | 4 (1-8) | 24.4 | 4 (1-8) | 24.3 |
| supports applications for research scholarships/ degrees | 4 (1-7) | 26.7 | 4 (1-7) | 26.7 |
| supports the peer-reviewed publication of research | 4 (1-7) | 28.0 | 4 (1-7) | 27.7 |
| Organisation level research skills/ success overall score | **4 (2-7)** | **23.6** | **4 (2-7)** | **23.6** |

**Q18,19 Barriers and Motivators for individual research** (RCC question except diversity question in barriers and sustainability question in motivators, both added by Champions)

| **Barriers** | **AHPs n (%)** | **All n (%)** |
| --- | --- | --- |
| Lack of time for research | 2522 (80.6) | 2673 (80.4) |
| Lack of suitable backfill | 1608 (51.4) | 1686 (50.7) |
| Other work roles take priority | 2606 (83.3) | 2764 (83.1) |
| Lack of funds for research | 1519 (48.6) | 1606 (48.3) |
| Lack of support from management | 1261 (40.3) | 1355 (40.8) |
| Lack access to equipment for research | 836 (26.7) | 887 (26.7) |
| Lack of administrative support | 1479 (47.3) | 1566 (47.1) |
| Lack of software for research | 1001 (32.0) | 1058 (31.8) |
| Isolation | 507 (16.2) | 535 (16.1) |
| Lack of library/internet access | 252 (8.0) | 271 (8.2) |
| Not interested in research | 308 (9.8) | 324 (9.7) |
| Other personal commitments | 1009 (32.1) | 1049 (31.5) |
| Desire for work / life balance | 1681 (53.7) | 1763 (53.0) |
| Lack of a co-ordinated approach to research | 930 (29.7) | 992 (29.8) |
| Lack of skills for research | 1384 (44.2) | 1429 (43.0) |
| Intimidated by research language | 954 (30.5) | 976 (29.4) |
| Limited by fear of getting it wrong | 858 (27.4) | 886 (26.6) |
| Lack of diversity and inclusion in research | 157 (5.0) | 176 (5.3) |
| Other | 139 (4.4) | 147 (4.4) |
| Motivators | **AHPs n(%)** | **All n (%)** |
| To develop skills | 2494 (80.7) | 2634 (80.2) |
| Career advancement | 1635 (52.9) | 1730 (52.6) |
| Increased job satisfaction | 1929 (62.4) | 2057 (62.6) |
| Study or research scholarships available | 580 (18.8) | 607 (18.5) |
| Dedicated time for research | 1330 (43.0) | 1411 (42.9) |
| Research written into role description | 821 (26.6) | 873 (26.6) |
| Colleagues doing research | 826 (26.7) | 871 (26.5) |
| Mentors available to supervise | 1262 (40.8) | 1327 (40.4) |
| Research encouraged by managers | 1116 (36.1) | 1187 (36.1) |
| Grant funds | 857 (27.7) | 910 (27.7) |
| Links to universities | 1166 (37.7) | 1239 (37.7) |
| Forms part of post graduate study | 716 (23.3) | 755 (23.0) |
| Opportunities to participate at own level | 1133 (36.7) | 1195 (36.4) |
| Problem identified that needs changing | 1668 (54.0) | 1768 (53.8) |
| Desire to prove a theory / hunch | 1199 (38.8) | 1274 (38.8) |
| To keep the brain stimulated | 1570 (50.8) | 1681 (51.2) |
| Increased credibility | 1226 (39.7) | 1280 (39.0) |
| Desire to improve sustainability (environmental, societal, economic) | 880 (28.5) | 940 (28.6) |
| Other | 205 (6.6) | 216 (6.6) |
