# Supplementary material for: Allied health professionals’ perceptions of research in the United Kingdom national health service: a survey of research capacity and culture
Source: BMC Health Serv Res. 2022 Aug 27;22:1094. doi: 10.1186/s12913-022-08465-6 (PMC9420271; doi:10.1186/s12913-022-08465-6)
Supplement: Supplementary file 3 — Additional file 3. Infographic summary of survey and key findings. [file 12913_2022_8465_MOESM3_ESM.pdf]

AHP Research Survey

cahpr Council for Allied Health Professions Research

NIHR | National Institute for Health Research

Research is everyone's business... isn't it?

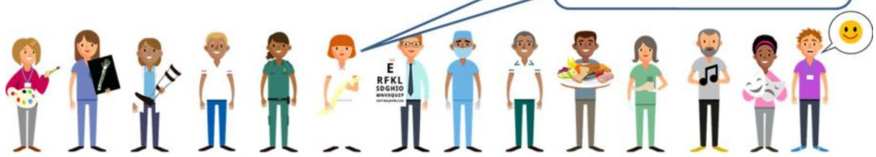

Reproduced with kind permission HEE © 2020

3145

AHPs working across the UK in NHS health and social care took part in the survey

Why this study is important

- AHPs play a key role in meeting escalating healthcare demands
- Research is the single most important way to improve healthcare
- AHP-led research evidence is needed to underpin advancing AHP roles
- Little is known about current AHP research capacity or culture

RESEARCH CAPACITY AND CULTURE

Motivators for research engagement

81% developing skills

62% increased job satisfaction

Barriers to research engagement

83% prioritisation of other job roles

81% lack of time

34% research included in job role → **21%** of these have ≥25% time allocated for research

18% routinely discuss research at appraisal

RESEARCH RATINGS

Individual research skills/success

Team research skills/ success

Organisation research skills/success

Less than adequate

RECOMMENDATIONS

national strategic

improve visibility of research organisations; ensure they are relevant to and provide support targeted at AHP clinical managers; set expectations for a stronger AHP research culture in NHS health and social care teams

organisation level

include a focus on AHP research posts and career pathways in research strategy; support middle managers in implementing the organization's research strategy

team level

introduce routine discussions focusing on research engagement during appraisals; capitalise on positive benefits from research (development of skills, job satisfaction) that are likely to impact on staff recruitment and retention

Individual level

build on individual motivation; consider using a self-appraisal tool to identify research development needs and aspirations

Christine Comer, Richard Collings, Alison McCracken, Carol Payne, Ann Moore 2022
